# The clinical and immunological performance of 28 days survival model of cecal ligation and puncture in humanized mice

**DOI:** 10.1371/journal.pone.0180377

**Published:** 2017-07-17

**Authors:** Krzysztof Laudanski, Natalia Lapko, Mateusz Zawadka, Benjamin X. Zhou, Gwenn Danet-Desnoyers, George S. Worthen

**Affiliations:** 1 Department of Anesthesiology & Critical Care, University of Pennsylvania, Philadelphia, PA. United States of America; 2 Faculty of Medicine, Ivano-Frankivsk Medical Institute, Ivano-Frankivsk, Ukraine; 3 2nd Department of Anesthesiology and Intensive Care, Medical University of Warsaw, Warsaw, Poland; 4 Technical College of New Jersey. Ewing, NJ. United States of America; 5 Department of Medicine, University of Pennsylvania, Philadelphia, PA. United States of America; 6 Department of Pediatrics, Children’s Hospital of Philadelphia, Philadelphia, PA. United States of America; University of Kentucky, UNITED STATES

## Abstract

Sepsis triggers a coordinated and thorough immune system response with long-term unfavorable sequelae after the initial insult. Long-term recovery from sepsis has garnered increasing attention recently, but a lack of suitable animal models impairs progress in this area. Our study, therefore, aimed to address the performance of the immune system in a survivable model of sepsis (cecal ligation and sepsis; CLP) for up to 28 d after the initial injury in humanized mice. Our model mimics human sepsis with weight loss and post-sepsis hypothermia. Within the first 7 d of sepsis, the M1 inflammatory cell subtype predominated, as evidenced by increased CD16 expression, but at 28 d, a mixed population of M1 and M2 inflammatory cells emerged, as evidenced by increased secretion of transforming growth factor TGFβ and CD206 expression. This change was accompanied by normalized production of interleukin (IL)-6, tumor necrosis factor TNFα and IL-10 at 28 d. Furthermore, the ability of MO to become regulatory DC or the frequency of endogenous DC were severely affected at 28 days. Thus, sepsis results in profound and persistent changes in the function of myeloid cells up to 28 days after CLP demonstrating the persistence of the new acquired immunological features long after resolution of the sepsis.

## Introduction

Long-term outcomes of sepsis have been the focus of increasing attention [1,2,3]. Although animals models proved to be invaluable in advancing our understanding of sepsis, inherent limitations of these models render a translation of findings in these models into clinically significant improvements in mortality and morbidity quite difficult [4]. Differences in immunology between humans and mice, inbreeding, and the relevance of the animal models to common clinical scenarios are frequently mentioned as obstacles [4,5,6]. On the other hand, research involving human subjects requires the utmost attention to patient well-being and ethical standards. Human trials are also affected by high inter-individual heterogeneity and lack of accounting for the pre-existing immunological make-up of the patients. Humanized mice have been proposed as an alternative to bridge the divide between animal studies and clinically trials [7,8].

Early use of antibiotics and aggressive source control has been credited with the reduction of early mortality in sepsis [1,3,9]. Consequently, more individuals survive the initial phase of the septic response. However, little is known about the performance of the immune system in septic survivors [1,4,9,10,11,12]. There is a clear need for a development of an animal model of sepsis that mimics clinical situation and long-term survival. Humanized mice have been found to share several characteristics with prior animal models and clinical responses in the early phase of sepsis, but no study addressed long-term performance of this model after the acute phase of an experimental septic shock [7,13,14].

Here, we investigated the long-term effects of sepsis and septic shock employing the well-established model of cecal ligation and puncture (CLP) in humanized mice [15]. We specifically assessed a performance of the myeloid system, which is critical during the transition from the initial innate immune response to acquired immunity and healing, in this process [4,5,6,16,17,18].

## Materials and methods

### Humanized mice and the long-term CLP survival model

The study was carried out in strict accordance with the recommendation of the Guide for the Care and Use of Laboratory Animals of the National Institute of Health. The Institutional Animal Care and Review Committee at the University of Pennsylvania (Philadelphia, PA) approved this study (protocol#830610). All animals were adequately anesthetized during procedures and 1ml of 0.5% bupivacaine was given to ameliorate the pain after surgery. Animals were monitored at least daily by research staff in addition to regular twice a day checks by members of an animal facility. Because we were interested in animal survival, we had to choose late endpoints for euthanasia (animal not moving, no food or water intake, agonal breathing, a temperature below 30°C).

Sublethally irradiated (3 Gy/mouse) NOS scid gamma (NSG) mice were transplanted with human umbilical cord blood CD34^+^ cells (1.5×10^5^ cells/mouse) intravenously as in other humanized model[7,14]. This is the minimally pain-related procedure. The adequacy of human immune reconstitution was assessed in peripheral blood using flow cytometry at 8, 12, and 16 weeks after the CD34^+^ stem cell transplantation. Animals were randomized into several groups at the beginning of the procedure: control (CONTR; n = 13) sham operated (SHAM; n = 16), and CLP. The remaining post-CLP pool of mice was further randomized to be sacrificed 24 h, (CLP_+24h_; n = 8), 7 d (CLP_+7d_; n = 8), and sacrifice 28 d (CLP_+28d_; n = 36).

The CLP procedure was performed according to the classical description by Chaudry and colleagues [15]. Animals were anesthetized via inhalation of isoflurane, which was titrated to achieve the desired effect. After sterile preparation with 70% isopropyl alcohol and 2% betadine, a midline incision through the skin, fascia, and the abdominal muscle were performed. The cecal appendage was visualized, and a reabsorbing suture was placed at the flexure and gently tightened. Next, two punctures were performed using a 25G needle, and a small amount of fecal material was gently squeezed out, ensuring that the cecum had been breached. The fascia and skin were closed with separate series of sutures, and 0.5 ml of 0.25% bupivacaine was injected along the suture line to provide analgesia. In SHAM animals, all the above steps were performed except ligation and perforation of the cecum. The animals were maintained on a pad heated to 37°C at all times. The surgery was performed under an infrared lamp with a 40-W bulb positioned approximately 30 cm from the animal to prevent intra-operative hypothermia. We used regional, long-term anesthetics instead (0.5% bupivacaine injected intradermally in the wound suture). Then, 50μl/g of pre-warmed 0.9% aqueous solution of NaCl was subcutaneously injected in a scarf to hydrate animals every 24 h for 5 d. Antibiotics (12.5 mg/kg metronidazole and 25 mg/kg ceftriaxone) were administered intraperitoneally every 12 h for a total of 5 d. Antibiotics were dissolved in 0.9% of aqueous solution of NaCl and usual injected volume was 1ml. This process was designed to mimic “typical sepsis” progression and therapy [19].

3 hours after surgery animals were assessed to ensure well-being. Increased rigid tenderness, abdominal distention, lack of willingness to drink water was considered indicative of intestinal obstruction, and such animals were euthanized at 3 hours mark.

Post-procedure animals were assessed daily for mortality beginning 24 h after CLP for a total of 28 days. Weights were noted before CLP and 7 and 28 d after CLP. Temperatures were measured with an infrared thermometer 6 and 18 h after surgery. Access to water and food (gel diet) was *ad libitum*. A standard day/night cycle was maintained throughout. Animals were kept in a sterile facility behind an aseptic barrier. The environment was maintained by the Animal Facility associated with the Veterinary School at the University of Pennsylvania.

Euthanasia was performed by placing an animal in specially designed Plexiglas tank and slowly increasing CO_2_. One minute after an animal stopped breathing stopped a cervical dislocation ensured death.

### Isolation of myeloid cells and functional assessment

Myeloid cells were collected using a magnetic bead separation technique as described previously [18,20]. Cytokine production was measured in 2×10^4^ myeloid cells in 200 μl of X-VIVO 10/15™ media with gentamycin (10 μg/ml), and phenol red (Lonza; Cohasset, MN) supplemented with 100 ng/ml lipopolysaccharide (lipopolysaccharide (LPS); Sigma-Aldrich, St. Louis, MO). Cells were incubated for 24 h [20]. The ability of monocytes (MO) to become dendritic cells (DC) was measured as previously described[20].

### Cytokine and serum marker measurements

Cytokine (Tumor Necrosis Factor α (TNFα), interleukin 6 (IL-6), interleukin 10 (IL-10), granulocyte monocyte colony stimulation factor (GM-CSF) levels in supernatants were measured using a magnetic multiplex kit (Bio-Rad; Hercules CA) according to the manufacturer’s protocol and analyzed on the BioRad™ platform.

### Statistical analysis

The parametric nature of the data was confirmed using the Levene’s and Shapiro-Wilk tests. Parametric data were presented as the average ± standard deviation (SD) while non-parametric variables were denoted as media (M_e_) with 25–75% interquartile range (IQ). The *t*-tests and Wilcoxon matched pair tests were conducted depending on the characteristics of the data. ANOVA and Friedman tests were also employed. A Duncan test was used for posthoc analysis. A *p-*value ≤ 0.05 was considered statistically significant. Statistica v8.0 (Statistica, Tulsa, OK) was used for statistical analysis.

Data has been included as Supplementary Materials.

## Results

### Physiological response of humanized mice mimics that of septic patients

**Fig 1:**
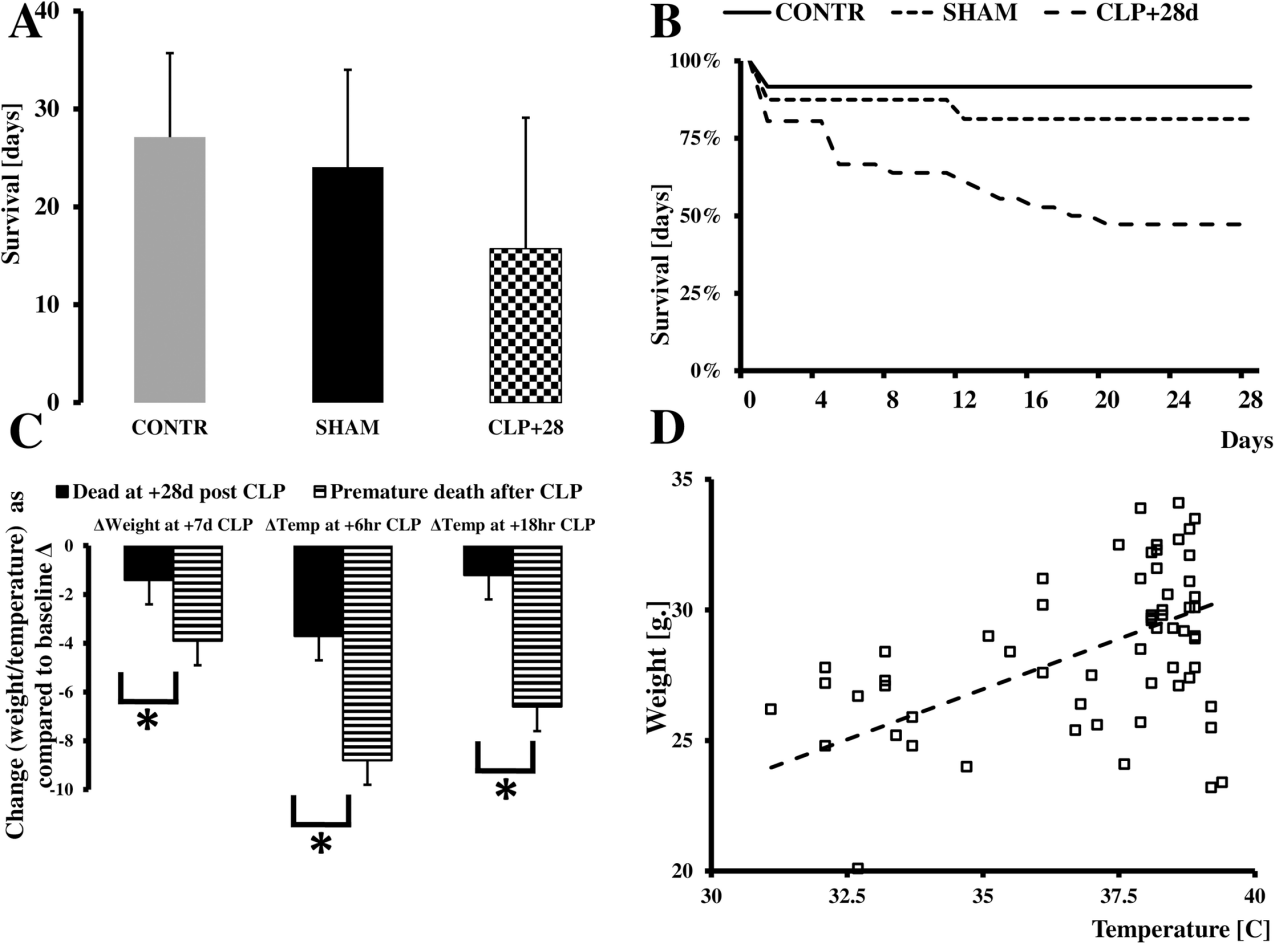
Natural history of CLP in humanized mice. We observed a significant decrease in animal mortality (A). Most deaths occurred either shortly after surgery or two weeks after the initial insult. Animals who died prematurely exhibited a significant drop in temperature at 6 (p = 0.0043) and 18 h (p = 0012) after CLP, while survivors were able to regain their core temperatures, at least partially (C). Also, the drop in weight was more pronounced in animals dying prematurely (p = 0.025) (C). A strong correlation was observed between drop in temperature at six h and weight loss post-CLP (*r*^*2*^ = 0.37;p = 0.023) (D).

Animals undergoing CLP had significantly increased mortality at 28 d compared to the SHAM or CONTR animals with survival rates of 47%, 81%, and 92%, respectively (Fig 1A and 1B; Supporting file S1 DATABASE). A significant number of these deaths occurred in the second week following CLP.

All animals undergoing CLP exhibited a significant drop in temperature six h after surgery as compared to CONT or SHAM mice, despite aggressive temperature control during the surgery.

Finally, we observed recovery of some of the function. We focused on kidney function and spleen structure. We found that serum level of creatinine increased initially to somewhat normalized after 28 days but marked diversity of recovery was apparent (Crea_CONT_ = 7.1±2.67; Crea_SHAM_ = 6.1±2.02; Crea_t+24hr_ = 32.4±8.75; Crea_t+7dr_ = 13.4±4.7; Crea_t+28d_ = 16.1±10.89; p = 0.003).

The mice had partially recovered from hypothermia 18 h after surgery (Table 1; Supporting file S1 DATABASE). Also, a significant body weight loss in CLP animals was observed 7 and 28 d after surgery (Table 1; *p* = 0.0026). When we compared these post-CLP changes between surviving and non-surviving animals, we noted several interesting differences. First, animals that died prematurely had a much more profound drop in body temperature at six hour (*p* = 0.0043) and 18 h (*p* = 0.0012) after surgery, and these mice also lost significantly more body weight in the 7 d following surgery (*p* = 0.0025; Fig 1C). A significant correlation between the drop in body temperature at 6 (Fig 1D; *r*^*2*^ = 0.56; *p*<0.0054) and 18 h (data not shown; *r*^*2*^ = 0.37; *p*<0.023) and weight at 7 d was detected.

**Table 1:** Changes in temperature and weight as compare to the time before the surgery.

	+6hrs			+18hrs			+7days			+28days		
	CONTR	SHAM	CLP	CONTR	SHAM	CLP	CONTR	SHAM	CLP	CONTR	SHAM	CLP
**Temp** **change [X±SD]**	0.06±0.46	-0.51±1.07	-6.54±2.67*	0.17±0.4	-0.01±0.43	-3.41±6.13*						
**% Weight change [X±SD]**							98±3	101±7	89±6*	102±1	103±4	89±5*

*denotes significant difference in comparison to CONTR

### Myeloid population in humanized population mimics long-term clinical events

**Fig 2:**
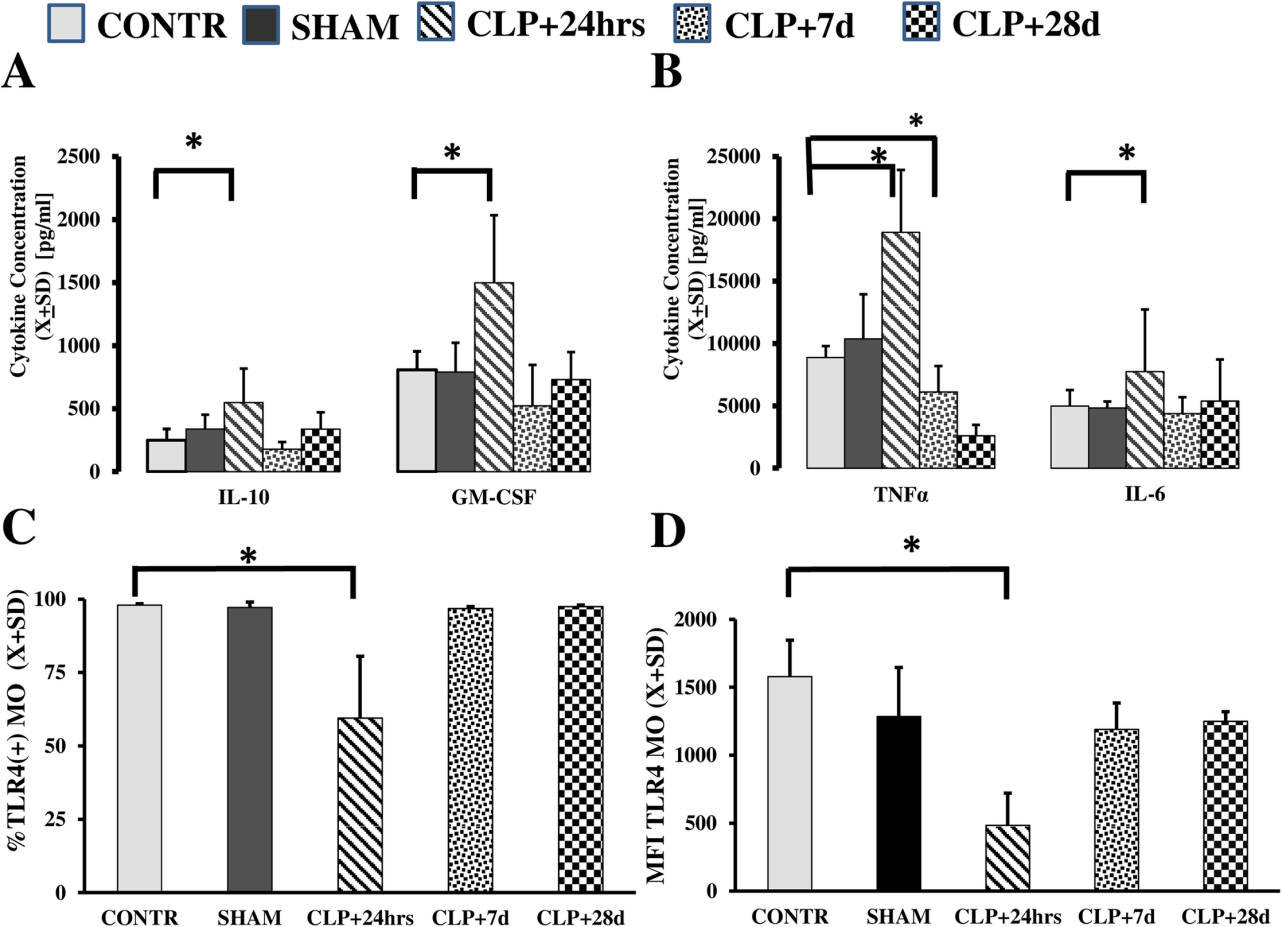
Cytokine production following CLP exhibits a time-dependent pattern. In the posthoc analysis, MO stimulated with LPS responded with increased production of IL-10 (*p* = 0.041) and IL-6 (*p* = 0.003) only at 24 h after CLP. TNFα secretion was increased at t+24 h (*p* = 0.00001) followed by suppression of TNF production at t+7 d (*p* = 0.026). After 28 d (t+28 d), production of these cytokines returned to pre-CLP status. Production of GM-CSF was not significantly increased at any time point. Expression of the TLR4 receptor was depressed at t+24 h regarding the percentage of positive cells (*p* = 0.003) and receptor density (*p* = 0.0008).

We then examined the responsiveness of the harvested myeloid cells to LPS at 24 h, 7 d, and 28 d after CLP. Each of the studied cytokines displayed different dynamics over time (Fig 2A and 2B). Secretion of GM-CSF (*p* = 0.039), TNFα (*p* = 0.0011), and IL-6 (p = 0.0012) increased at 24 h, but at seven days and later the levels dropped to the pre-CLP levels. Secretion of IL-10 was not significantly increased at any time after CLP (*p* = 0.19). Because we used LPS to stimulate the cells, we measured the expression of Toll-like receptor 4 (TLR4) and CD14 receptors. Expression of the TLR4 receptor was transiently decreased in both a frequency of positive cells (*p* = 0.033) and surface receptor density on circulating myeloid cells (p<0.023).

**Fig 3:**
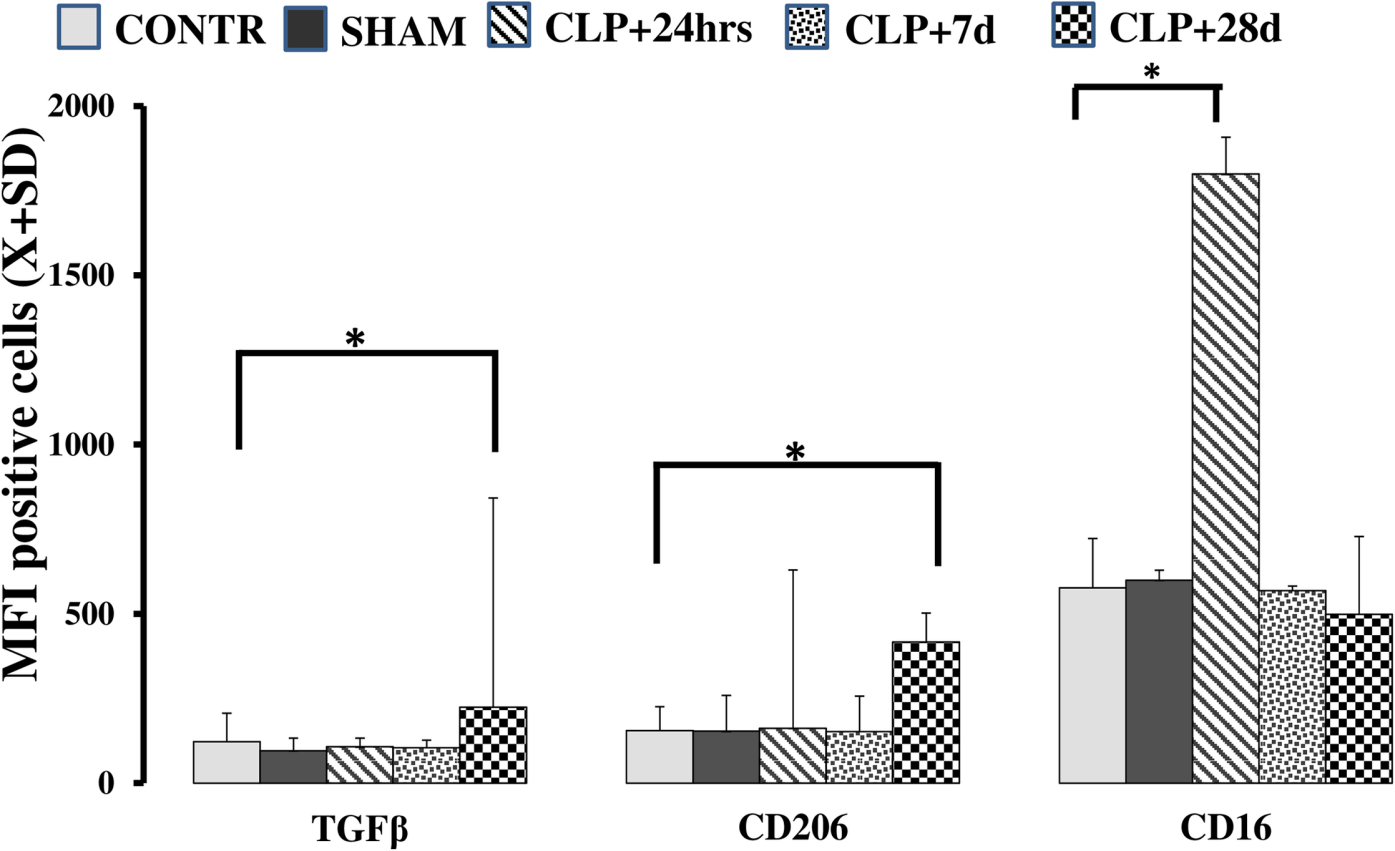
Flow cytometry in MO after CLP suggests an M1-to-M2 shift. Post-CLP, a late increase in expression of TGFβ/LAP (Mean Fluorescence Intensity (MFI); *p* = 0.047) and CD206 (*p* = 0.049) was seen. In contrast, expression of CD16 was increased only shortly after CLP MFI (*p* = 0.022) compared to pre-CLP expression levels.

In the next step, we observed changes in the composition of peripheral blood MO markers. This flow cytometry technique is an established method for the characterization of myeloid cells using surface markers [16,21,22]. Immediately after surgery (t+24 h), the frequency of CD16^+^ cells rapidly increased (Fig 3;*p* = 0.004). Furthermore, the expression of both CD206 (*p* = 0.005) and transforming growth factor TGFβ (*p* = 0.002) was elevated compared to levels in CONTR animals that survived for 28 d post-surgery (Fig 3A and 3B). Finally, expression of the receptor for monocyte colony-stimulating factor (M-CSF R), a macrophage marker, was significantly increased 28 d after CLP in surviving mice (CONTR_CD115_ = 2091.6±443.59; SHAM _CD115_ = 2300.5±388.52; CLP _CD115_ = 6906.1±3347.58; *p* = 0.0021).

### Plasticity of MO to develop into DC is limited post-CLP

**Fig 4:**
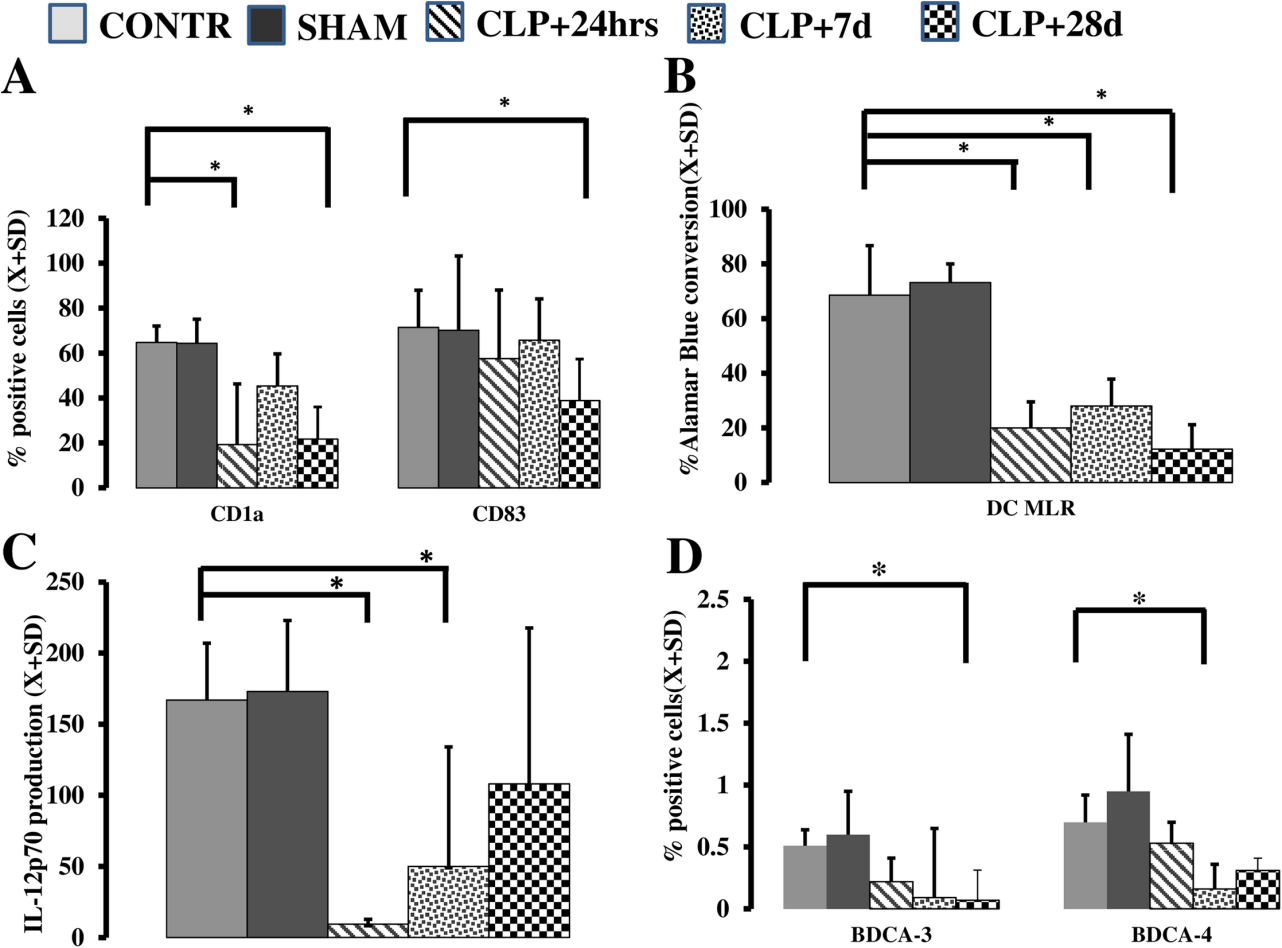
The ability of peripheral MO to become DCs is affected by CLP. An acquisition of CD1a marker on the MO differentiated with IL-4& GM-CSF showed significant depression at t+24 h (*p* = 0.0038) and t+28 d (*p* = 0.0030) (A). CD83 expression was depressed only 28 d after CLP (*p* = 0.048) (A). Functional ability of IL-4 and GM-CSF to stimulate allogeneic T cells (*p*<0.001) (B) and produce IL-12p70 (*p*<0.003) (C) was severely depressed following CLP. CLP caused a selective and profound depression of BDCA-3 (blood dendritic cell antigen type) endogenous DC 28 d post-surgery (*p* = 0.002) while frequency of BDCA-4^+^ cells was briefly depressed at 7d (*p* = 0.002) and recovered to pre-CLP levels (D).

Finally, we evaluated the ability of MO to become DC as a measure of developmental plasticity [6,16,22]. We stimulated cells obtained at various stages post-CLP insult with IL-4 and GM-CSF and evaluated the potential of these myeloid cells to differentiate into DC *in vitro*. Late (+28 d) after CLP, a significant decrease in the ability of MO to become DC was observed, as measured by diminished expression of CD1a (*p* = 0.003) and CD83 (*p* = 0.048; Fig 4A). These flow cytometry data were reinforced by functional data that demonstrated a low potential for MO from CLP animals to stimulate allogeneic T cells (*p*<0.001) (Fig 4B) and decreased expression of IL-12p70 (*p* = 0.003) (Fig 4C) 28 d post-surgery.

We also detected a significant decrease in frequency of blood dendritic cell antigen BDCA-3^+^ cells (*p* = 0.002) as well as BDCA-4^+^ cells (*p* = 0.009) in peripherally circulating leukocytes 7 and 28 d post-surgery; however, the population of BDCA-2^+^ cells was largely preserved (*p* = 0.12; Fig 4D; Supporting file S1 DATABASE) in peripherally circulating leukocytes at these time points.

## Discussion

Our research showed that humanized mice if treated with antibiotics and fluid resuscitation exhibit similar symptoms and survival as seen clinical situation. Profound changes in body temperature and severe weight loss, hallmarks of profound inflammatory response, were present in our model and correlated with early animal mortality. Shortly after induction of sepsis, the mice developed a significant decrease in core temperature, and this decline has been observed in other animal models [15,23,24]. In human subjects, hypothermia is less frequently found than fever at the onset of severe sepsis [23]. Also, septic animals had severe muscle wasting, as evidenced by the observed significant loss of body mass. Interestingly, the decreases in temperature and weight were correlated. Finally, acute renal failure was shown in our animals early. Abnormal thermoregulation and catabolism are hallmarks of the inflammatory response. Lack of control over the initial inflammation is often linked to mortality at this step [19,21,25].

Few long-term studies about the long-term performance of the immune system in humanized mice have been reported but none of them extend as long as ours [5,7,14]. In our experimental design, the late mortality was also significant. Its magnitude was similar as seen in a clinical situation [1,3,10,19]. Our study cannot answer why late mortality has occurred, but it is likely that progressive organ failure is the underlying cause. In our study, the recovery of the kidney function was highly diverse suggesting that in animals with demise before 28 days had progressive organ failure.

Our model not only mimics the natural history of peritonitis but also allows for long-term surveillance of the mortality of the mice. Furthermore, the introduction of early antibiotic and fluid resuscitation not only result in a decrease in early mortality but also parallel the typical treatment of sepsis in the much more clinical relevant way [19,26]. Our goal was not to compare humanized mice to existing mice model of CLP but rather observe the natural history of CLP sepsis in xenotransplanted mice and describe the evolution of the immune system over time.

In our analysis of the myeloid cells from the humanized mouse model of sepsis, we found a significant change in the performance of MO after induction of a septic episode. Initially, secretion of several cytokines (TNFα and IL-6) was significantly increased with a shift toward an inflammatory M1 cell type, which is characteristic of increased CD16 expression in agreement with prior findings suggesting a systemic inflammatory response early after insult [11,21,27]. An early increase in production of several cytokines is frequently found in sepsis, as the MO are primed by the initial insult to release even more cytokines when re-exposed to the pathogen [12,14]. Our flow cytometry data suggest a clear shift of the MO population from M1 to M2 between 7 and 28 d post-CLP of the MO population from M1 to M2 [10,17,27]. The emergence of M2 cells is usually related to the appearance of compensatory anti-inflammatory response syndrome (CARS) and gradual extinguishing of the inflammatory episode. Yang *et al*. suggested that M-CSF may play a critical role in this shift [28]. In conclusion, our study is the first report of an M2 shift in CLP humanized mice surviving sepsis.

The capability of MO to differentiate into professional dendritic cells (DC) as very complex. We did not observe any decrease in the expression of CD83 after sepsis onset. The expression of CD1a, production of IL-12p70, and the ability of MO to become DC were severely depressed early after CLP, suggesting accelerated maturation but failed acquisition of the functional maturity of the emerging DC as previously described [29]. After 28 d, all functional and cytometric markers of DC emergence were profoundly depressed, indicating that MO cannot effectively process signals to differentiate into DC long-term after sepsis [6,16,18]. This finding is well aligned with observation in other studies in the human subject. Moreover, not only MO-derived DC were depleted, but also the percentage of endogenous BDCA-3^+^ were significantly affected long-term after CLP. The preferential differentiation of circulating MO into M2 and simultaneous depletion of both MO-derived and endogenous of DC *in vivo*, suggest that bone marrow is the predominant source of post-septic long-term leukocyte aberrations [11,27,30]. Considering that some of the newly acquired features of MO persevered longer than their lifespan our study offers an opportunity to look into bone marrow changes in the human-centered model but unclear dynamics between mice post-radiation mesenchymal cells, and human bone marrow progenitors introduce significant unknown. Furthermore, prolongation of the defect beyond initial insult suggests that the resolution of inflammation may take longer than initially thought. Smoldering inflammation leads to the progressive organ damage and may be responsible for late mortality.

Our model has to be looked at with several limitations in mind. We performed cecal perforation followed by induced ischemia without repair. This is not a typical medical practice. In the relevant clinical scenario, a remedial surgery would be conducted. Here, we relied on the innate ability of rodents to tolerate peritonitis, as well as the ability of peritoneum to wall off the infection. Also, we do not know to what extend the gut microflora in humanized mice mimics flora of humans [31]. In the optimal model, we would take mice with germ-free and inoculate with human intestinal flora[5,7,8,14]. Human gnotobiotic mice would an optimal model but even such model is questioned since gut flora, and immune system do not co-develop in humanized mice model[31]. Also, reconstruction of mesenchymal bone marrow support in humanized mice is impaired as well. Thus, production of cytokines necessary for bone marrow activation during sepsis is not adequate to the needs of transplanted human cells. On the other hand, eicosanoid acid derivatives share similar structures between human and mice. Under optimal circumstances, we would compare the response in human induced bowel perforation, or in native irradiated mice with subsequent autologous bone marrow transplant to the humanized model of CLP. However, such experiments are unethical and not devoid of the methodological flaws (e.g. how to discern the effect of transplantation or radiation itself on CLP, are NSG mice representative of other mice species?). This study was intended as a pilot for the performance of humanized model, not a study pinpointing the effect of mice “humanization” itself. Considering that it is unknown how xenotransplanted immune system interacts with nervous, endocrine, and other systems, which are of mice origin, we decided not to analyze the interaction between several internal organs and system but focus on the performance of the immune system. Finally, we did not aim to predict which animals would die. Restriction related to exposing animals to visitors, lack of wireless physiological monitoring, and the inability of humanized mice to withstand a small amount of blood draw if septic prevented us from investigating the most immediate causes of death in the surviving animals.

## Conclusions

In summary, our study demonstrated that a modified CLP humanized mouse model of sepsis recapitulates the physiological and epidemiological characteristics of human sepsis quite well. Furthermore, we revealed a significant M1→M2 shift that was accompanied by a decline in the ability of MO to differentiate to DC both *in vitro* and *in vivo*. These changes were typical for late phase of emergence from sepsis and suggest an inability of the immune system to control itself.

## Supporting information

S1 DATABASE Database of the vital signs, cytokine production and flow cytometry data. (XLSX)
